# Downregulation of inhibitor of apoptosis-stimulating protein of p53 inhibits proliferation and promotes apoptosis of gastric cancer cells

**DOI:** 10.3892/mmr.2015.3587

**Published:** 2015-04-01

**Authors:** LU-LU WANG, ZHONG XU, YANG PENG, LU-CHUN LI, XIAO-LING WU

**Affiliations:** 1 Department of Gastroenterology, Second Affiliated Hospital of Chongqing Medical University, Chongqing 400010, P.R. China; 2 Department of Gastroenterology, Guizhou Provincial People’s Hospital, The Affiliated People’s Hospital of Guiyang Medical University, Guiyang, Guizhou 550002, P.R. China

**Keywords:** inhibitor of apoptosis-stimulating protein of p53, gastric cancer, apoptosis

## Abstract

Gastric cancer (GC) remains one of the leading causes of cancer-associated mortality. Inhibitor of apoptosis-stimulating protein of p53 (iASPP) is a member of the inhibitory apoptosis-stimulating protein p53 family. The overexpression of iASPP has been detected in several types of tumor in humans. However, the role of iASPP in GC remains to be elucidated. The objectives of the present study were to detect the expression of iASPP in GC and examine the potential role of iASPP in GC cell lines. Using reverse transcription-quantitative polymerase chain reaction and western blot analyses, it was identified that the expression of iASPP in GC tissues and GC cell lines was higher compared with that in adjacent normal tissues and in a normal gastric mucosa cell line (GES-1). To examine the role of iASPP in GC cells, the expression of iASPP was inhibited using a small interfering (si)RNA against iASPP and it was observed that iASPP expression was significantly downregulated. Using MTT assays, colony-formation assays and flow cytometry, it was identified that the inhibition of iASPP was able to significantly inhibit the proliferation and colony forming ability and promote apoptosis in GC cells. To examine the role of iASPP in GC cells *in vivo*, GC cells, which were infected with iASPP-siRNA or control-siRNA were subcutaneously injected into nude mice. It was identified that downregulation of iASPP significantly inhibited tumor growth *in vivo*. Thus, iASPP may be a potential molecular target in GC therapy.

## Introduction

Gastric cancer (GC) remains a significant threat to human life, although significant progress in the diagnosis and treatment of this disease has been achieved (1). As with all solid tumors, GC is thought to initiate and progress through a series of genetic alterations. Recently, increasing attention has been focused on gene therapy.

Inhibitor of apoptosis-stimulating protein of p53 (iASPP) acts as a negative regulator of p53 function, inhibiting p53 by directly binding to its DNA-binding domains (2,3). The p53-binding region of iASPP has a certain similarity with that of its other family members, p63 and p73 (4). Previous studies have revealed that iASPP may also interact with p63 and p73 and affect their function (5). Furthermore, iASPP, also known as RelA-associated inhibitor, is able to regulate the function of nuclear factor-κB (NF-κB) (6,7).

iASPP, the only homologue of the ASPP family, was identified as an oncogene by detection of abnormal overexpression of iASPP in several types of human cancer, including breast carcinomas (8,9), acute leukemia (10), lung cancer (11) and hepatocellular carcinoma (12). These data indicated that iASPP may be important in the development of tumors in humans. However, only a few studies have investigated the role of iASPP in human GC (13). In the present study, the expression of iASPP in GC tissues and GC cell lines was analyzed and then the potential role of iASPP in the GC cell lines was examined *in vivo* and *in vitro*.

## Materials and methods

### Tissue samples and immunohistochemistry

GC tissue (46 samples) and the adjacent normal gastric mucosal tissue (30 samples) were collected from patients who underwent surgery at the Second Affiliated Hospital of Chongqing Medical University (Chongqing, China) between September 2012 and March 2014. All GC tissues were confirmed by pathological examination, and the adjacent normal gastric tissues were obtained from 5 cm away from the GC tissues. Informed consent was obtained from the patients and the present study received approval from the Institutional Review Board of the Second Affiliated Hospital of Chongqing Medical University. The present study was conducted in accordance with the ‘Biomedical Research Involving Human Ethics Review (Tentative)’ regulation of the Ministry of Health and the Declaration of Helsinki on Ethical Principles for Medical Research Involving Human Subjects.

The expression of iASPP protein in the samples was detected using immunohistochemistry. The GC tissue and adjacent normal gastric mucosal tissue samples were paraffin-embedded (Sigma-Aldrich, St. Louis, MO, USA) and 4-*µ*m sections were prepared. The sections were incubated with 3% H_2_O_2_ (Sigma-Aldrich) for 10 min at room temperature to eliminate endogenous peroxidase activity. Subsequently, the sections were incubated with monoclonal mouse iASPP antibody (ab49805; 1/1,000; Abcam, Cambridge, UK) for 90 min at 37°C and with a peroxidase-conjugated goat anti-mouse immunoglobulin IgG (SC-2005; 1:500; Santa Cruz Biotechnology, Inc., Dallas, TX, USA) for 20 min at room temperature. 3,3′-diaminobenzidine reagent (Sigma-Aldrich) was added onto each section, and subsequently, counter-staining was performed with hematoxylin (Sigma-Aldrich). The sections were then dehydrated in graded alcohol (50, 70, 85, 95 and 100%) and xylene (Sigma-Aldrich), cleared in distilled water and mounted with neutral gum (Bioworld Technology, Inc., St. Louis Park,. MN, USA).

### Cell lines

The GC cell lines (MKN45, BGC-823 and SGC-7901) were purchased from the American Type Culture Collection (Manassas, VA, USA). The GES-1 cell line was obtained from the Type Culture Collection of the Chinese Academy of Sciences (Shanghai, China). The GC cell lines were routinely maintained in RPMI 1640 medium supplemented with 10% fetal bovine serum (Gibco Life Technologies, Carlsbad, CA, USA) without antibiotics in a humidified atmosphere of 5% CO_2_ at 37°C. The GES-1 cell line was cultured in Dulbecco’s modified Eagle’s medium containing 10% fotal bovine serum (Gibco Life Technologies) and 10 mg/ml vancomycin (Santa Cruz Biotechnology, Inc.).

### Lentivirus transfection

Downregulation of iASPP was achieved by infecting the cells with the iASPP-small interfering (si)RNA lentivirus (Genepharma Co., Ltd., Shanghai, China). Target cells were plated in six-well plates at 20–30% confluence and incubated for 12 h prior to the infections with iASPP-small interfering (si)RNA- or scrambled control-siRNA-expressing lentiviruses (Genepharma Co., Ltd.). When the infections were performed, the culture medium was replaced with a supernatant fluid, which contained an appropriate viral titer (1 ml/well). After incubating at 37°C for 12 h, the viral supernatant was replaced with fresh media. The infected cells were selected using puromycin (2 mg/ml; Santa Cruz Biotechnology, Inc.) following incubation for 48 h. Successful infection was confirmed via expression of green fluorescent protein as confirmed using an inverted fluorescence microscope (Leica DMI4000 B; Leica Microsystems GmbH, Wetzlar, Germany). The knockdown efficiency was determined using western blot analysis.

### Reverse transcription-quantitative polymerase chain reaction (RT-qPCR)

Total RNA was extracted from the GES-1, MKN-45, SGC-7901 and BGC-823 cells using the RNAiso reagent (Takara Bio, Inc., Otsu, Japan) according to the manufacturer’s instructions. The RNA was reverse-transcribed into cDNA using the PrimeScript II First Strand cDNA synthesis kit (Takara Bio, Inc.). RT-qPCR was performed using LightCycler real-time PCR with the SYBR Premix Ex Taq™ kit for Perfect Real-Time (Takara Bio, Inc.). Primers were purchased from Takara Bio., Inc., and the sequences for PCR amplification of the iASPP gene were as follows: Forward, 5′-GCGGTGAAGGAGATGAACGA-3′ and reverse, 5′-TGATGAGGAAATCCACGATAGAGTAG-3′. The primer sequences for the internal control β-actin were as follows: Forward, 5′-CCACGAAACTACCTTCAACTCC-3′ and reverse, 5′-GTGATCTCCTTCTGCATCCTGT-3′. The PCR cycling conditions were as follows: 94°C for 60 sec, followed by 40 cycles of 94°C for 40 sec, 60°C for 40 sec and 6 min extension at 72°C. The relative gene expression levels were calculated using the 2^−ΔΔCT^ method.

### Protein preparation and western blotting

The target cells were washed twice with phosphate-buffered saline (PBS), harvested in radioimmunoprecipitation assay lysis buffer (Beyotime Institute of Biotechnology, Shanghai, China) and flash-frozen on dry ice. Following allowing the cells to thaw, the lysates were collected with a rubber scraper, sonicated and centrifuged at 12,000 × g (4°C for 20 min). The total protein concentration was measured using a Pierce bicinchoninic acid protein assay kit (Pierce Biotechnology, Inc., Rockford, IL, USA). To perform the western blot analysis, proteins were resolved using 10% SDS-PAGE (Bio-Rad Laboratories, Inc., Hercules, CA, USA) and transferred onto a polyvinylidene difluoride membrane (Merck Millipore, Darmstadt, Germany). Subsequently, the membranes were blocked for 1 h with 5% non-fat milk at room temperature and then incubated overnight at 4°C with the iASPP and β-actin primary antibodies. The secondary antibody was goat anti-mouse IgG conjugated to horseradish peroxidase (1:3,000). The signal was detected using an enhanced chemiluminescence reagent (EMD Millipore, Billerica, MA, USA). To analyze the iASPP protein levels, monoclonal mouse antibodies against the iASPP protein (828 amino acids, 92 kDa, 1:5,000, Abcam) were used. For the loading control, a monoclonal mouse β-actin antibody (A5441; 42 kDa, 1:5,000, Sigma-Aldrich) was used.

### Cell viability and colony formation assays

The effect of iASPP-siRNA on cell proliferation was detected using an MTT assay. The target cells were seeded into 96-well plates at a density of 1×10^4^ cells/well. An MTT solution (5 mg/ml MTT, 20 ml; Sigma-Aldrich) was added to the cultures (total volume of 200 ml) and incubated for 4 h at 37°C. Following removal of the culture medium, the remaining crystals were dissolved in dimethyl sulfoxide (Sigma-Aldrich) and the absorbance at 560 nm was measured using a Multiskan MK3 microplate reader (Thermo Fisher Scientific, Inc., Waltham, MA, USA).

The effect of iASPP-siRNA on the colony forming ability of the target cells was detected using a colony formation assay. To perform this assay, the target cells were seeded in six-well plates at a low density (1,000 cells/plate) and cultured until visible colonies appeared. The colonies were then stained with Giemsa stain (Santa Cruz Biotechnology, Inc.) and were counted.

### Detection of apoptosis

To further elucidate the association between iASPP and GC cells, the rate of cell apoptosis was determined using flow cytometry. The target cells were collected and washed twice with ice-cold PBS buffer. The apoptosis rate of cells was detected with an Annexin V-FITC Apoptosis Detection kit (eBioscience, Inc., San Diego, CA, USA) and propidium iodide (Sigma-Aldrich) double staining according to the manufacturer’s instructions. Flow cytometric analysis was performed using the BD LSRI flow cytometer (BD Biosciences, Franklin Lakes, NJ, USA) and data were analyzed using the CellQuest 5.1 software (BD Biosciences).

### Xenograft experiment

Male athymic nude mice (6–8 weeks old), were obtained from the Animal Experimental Centre of Chongqing Medical University. To establish the GC model, equal numbers of MKN-45 cells (1×10^6^) infected with iASPP-siRNA or control-siRNA lentivirus were injected subcutaneously into the right rear flank of each mouse (four mice per group). Tumor growth was observed daily in each group. The tumor volume was calculated as (LxS^2^)/2 where L is the longest tumor axis and S is the shortest tumor axis. At four weeks following injection, all mice were sacrificed via anesthesia using sodium pentobarbital (Sigma-Aldrich), then subjected to cervical dislocation, the xenografts were then resected from the mice and flash frozen in liquid nitrogen for further analysis. The present study was conducted in strict accordance with the recommendations of the Guide for the Care and Use of Laboratory Animals of Chongqing Medical University. The protocol was approved by the Committee on the Ethics of Animal Experiments of Chongqing Medical University. All surgical procedures were performed under sodium pentobarbital (Sigma-Aldrich) anesthesia and all efforts were made to minimize suffering.

### Statistical analysis

All experiments were repeated three times. Data were analyzed using SPSS 16.0 software (SPSS, Inc., Chicago, IL, USA). Values are expressed as the mean ± standard deviation. The statistical significance of the differences among the groups was evaluated using a t-test. P<0.05 was considered to indicate a statistically significant difference.

## Results

### iASPP is upregulated in GC tissues and cell lines

**Figure 1:**
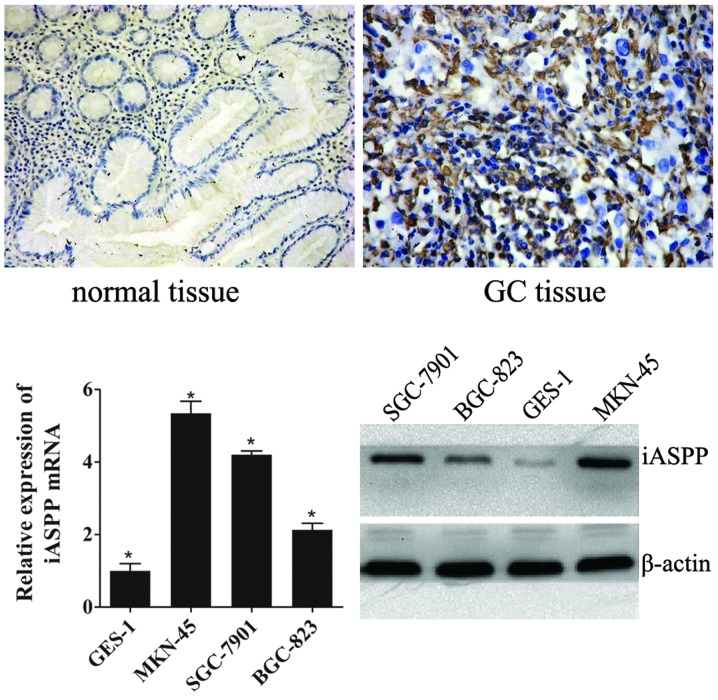
iASPP is upregulated in GC tissues and cell lines. iASPP expression was upregulated in GC tissues compared with that in adjacent normal tissues. iASPP mRNA levels were upregulated in three GC cell lines (MKN-45, SGC-7901 and BGC-823) compared with those in a normal gastric mucosa cell line (GES-1). iASPP protein levels were upregulated in the three GC cell lines compared with those in GES-1 cells. Magnification, x200. Values are expressed as the mean ± standard deviation; ^*^P<0.05. iASPP, inhibitor of apoptosis-stimulating protein of p53; GC, gastric cancer.

According to the results of the immunohistochemical analysis, the expression of iASPP in GC samples was significantly upregulated in GC tissues compared with that in their adjacent normal tissues (cells with brown staining in the cytoplasm or nucleus were regarded as iASPP-positive cells) (Fig. 1). According to the RT-qPCR and western blot analyses, the expression levels of iASPP were higher in the MKN-45, BGC-823 and SGC-7901 cell lines compared with those in the GES-1 cell line, illustrating that iASPP may be associated with the development of GC (Fig. 1). The expression of iASPP was higher in the MKN-45 and SGC-7901 cell lines compared with that in the other cell lines; therefore, these two cell lines were selected as the target cells for subsequent experiments. The MKN-45 cell line contains the wild-type p53 gene (14), whereas the SGC-7901 cell line carries a mutated p53 gene (15).

### Inhibition of iASPP expression inhibits proliferation and colony forming ability and promotes apoptosis in GC cells

**Figure 2:**
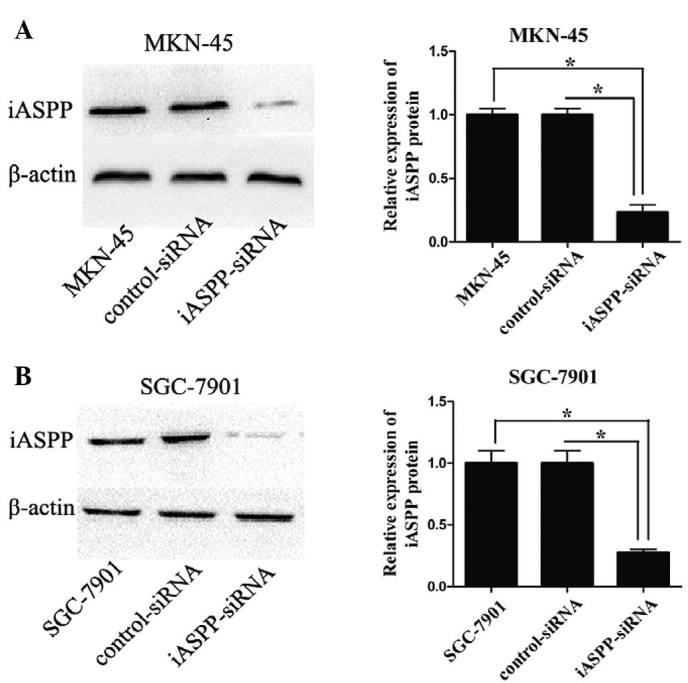
iASPP expression is significantly downregulated in gastric cancer cells following infection with iASPP-siRNA-lentivirus, while the control siRNA had no effect on iASPP protein levels. (A) iASPP expression in MKN-45 cells was inhibited following iASPP-siRNA-lentivirus infection. (B) iASPP expression in SGC-7901 cells was inhibited following iASPP-siRNA-lentivirus infection. Values are expressed as the mean ± standard deviation; ^*^P<0.05. iASPP, inhibitor of apoptosis-stimulating protein of p53; siRNA, small interfering RNA.

**Figure 3:**
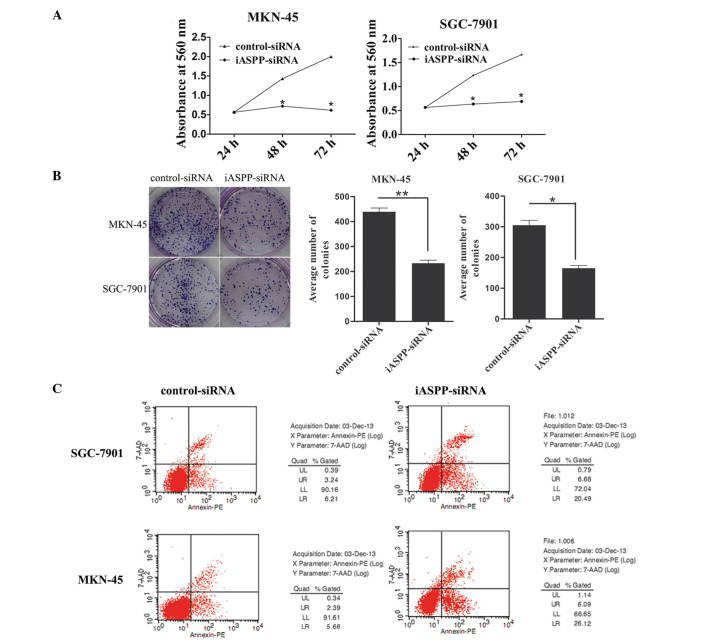
Effects of iASPP expression on GC cells. (A) An MTT assay indicated that GC cell proliferation was significantly inhibited following iASPP-siRNA-lentivirus infection. (B) Inhibition of iASPP expression inhibited the colony-forming ability of GC cell lines. (C) Inhibition of iASPP promoted GC cell apoptosis. Values are expressed as the mean ± standard deviation; ^*^P<0.05, ^**^P<0.01 vs. control. iASPP, inhibitor of apoptosis-stimulating protein of p53; siRNA, small interfering RNA; GC, gastric cancer; PE, phycoerythrin; UL, upper left; UR, upper right; LL, lower left; LR, lower right.

To examine the functional significance of iASPP in GC, GC cell lines (MKN-45 and SGC-7901) were infected with lentivirus containing iASPP-siRNA or scrambled control siRNA. Western blotting was performed to assess iASPP protein levels. Infection of cells with lentivirus containing iASPP-siRNA significantly reduced iASPP protein expression levels in the MKN-45 and SGC-7901 cells (Fig. 2). By contrast, the control siRNA had no effect on iASPP protein levels. As shown in Fig. 3, the decreased expression of iASPP reduced the proliferation and colony forming ability of cells. The iASPP-siRNA lentivirus-infected cells formed fewer colonies compared with the control-siRNA lentivirus-infected cells. Flow cytometric analysis revealed that decreased expression of iASPP enhanced the levels of cell apoptosis.

### Inhibition of iASPP decreases tumor growth in vivo

**Figure 4:**
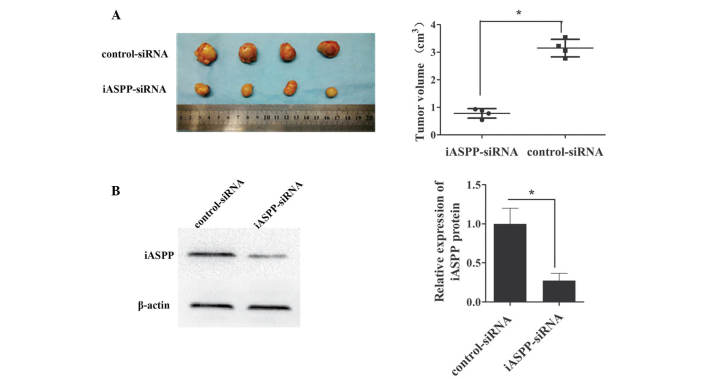
Inhibition of iASPP decreases tumor growth *in vivo*. (A) Growth of tumors of GC cells transfected with iASPP siRNA was significantly inhibited in a xenograft model compared with that of tumors of control siRNA-transfected cells. (B) iASPP expression was downregulated in tumors of GC cells transfected with iASPP siRNA. Values are expressed as the mean ± standard deviation; ^*^P<0.05. iASPP, inhibitor of apoptosis-stimulating protein of p53; siRNA, small interfering RNA; GC, gastric cancer.

To investigate the role of iASPP in tumor growth *in vivo*, nude mice were subcutaneously injected with an equal quantity of MKN-45 cells, which were transfected with iASPP-siRNA lentivirus or control-siRNA lentivirus (10^6^ cells/mouse). Tumors appeared in all mice. As shown in Fig. 4, forced downregulation of iASPP significantly inhibited tumor growth *in vivo*. iASPP expression in the xenograft tumors was measured using western blotting and it was identified that iASPP expression was significantly decreased in the tumor cells transfected with the iASPP-siRNA lentivirus as compared that in the control tumors.

## Discussion

iASPP is an evolutionarily conserved inhibitor of p53, and overexpression of iASPP has been observed in several types of human cancer (8–12). The present study examined GC cell lines and tumor samples to demonstrate that the expression levels of iASPP were higher in GC tissues and GC cell lines compared with those in their adjacent normal tissues and normal gastric mucosal cells. The present study suggested that abnormal expression of iASPP may be an important step in the development of GC and it may therefore be a useful molecular marker for the diagnosis of GC.

Li *et al* (16) observed that downregulation of iASPP is able to inhibit proliferation of the p53-mutant glioblastoma cell line U251. Zhang *et al* (17) demonstrated that a reduction of iASPP inhibited cell growth and induced apoptosis in p53-defective prostate cancer cells. Lin *et al* (18) reported that small hairpin RNA-mediated downregulation of iASPP repressed hepatocellular carcinoma cell proliferation and colony formation *in vitro* and inhibited the growth of tumors *in vivo*. Inhibition of iASPP also induced apoptosis in breast cancer cells (19). To the best of our knowledge, no studies have previously investigated the potential role of iASPP in the proliferation and apoptosis of GC cell lines. Therefore, in the present study, the expression of iASPP was inhibited via transfection with an iASPP-siRNA lentivirus. Following the transfection, the proliferation, colony formation and apoptotic rate were assessed. The results revealed that following the downregulation of iASPP expression using iASPP-siRNA, the two cell lines exhibited a reduction in the proliferation and colony forming ability. This indicated that knockdown of iASPP is able to significantly inhibit the growth of GC cells and may therefore be a useful approach for anti-tumor therapy. In addition, knockdown of iASPP expression induced apoptosis in the two GC cell lines, which indicates an oncogenic function of iASPP, as an imbalance between proliferation and apoptosis contributes to the formation and development of human tumors (20). Additionally, an *in vivo* xenograft experiment identified that tumor growth was significantly inhibited by knockdown of iASPP. Thus, it was concluded that iASPP may act as a potential oncogene in GC and that iASPP may be an effective target in the treatment of GC.

p53 is critical in apoptosis, having a high frequency of mutations in various types of human cancer (21). However, mutations in the p53 gene do not appear to be a necessary event in human carcinomas. Wild-type p53 is retained in ~50% of human tumors (22); however, its tumor suppressive function appears to be inhibited in tumor cells. The identification of the ASPP family provided novel insight into the mechanism underlying the suppression of p53 activity in cancer cells. In the present study, overexpression of iASPP in the p53 wild-type MKN-45 cell line inhibited the apoptotic function of p53, promoting the progression of GC. The present study revealed that expression of iASPP in p53 mutant SGC-7901 cells was also upregulated. However, iASPP is unable to interact with the mutated form of p53 (8). This finding raises questions regarding the mechanism of action of the iASPP gene. iASPP has been observed to bind to the NF-κB subunit RELA/p65 and inhibit its transcriptional activity, which has important roles in the control of cell proliferation and apoptosis (6,7). Furthermore, iASPP may also interact with p63 and p73 and affect their functions (4). Dissimilar to p53, p63 and p73 are not commonly mutated in human tumors (23,24).

In conclusion, the present study suggested that iASPP may have an oncogenic function in GC. The results also indicated that inhibition of iASPP is important in the downregulation of cell proliferation and the activation of apoptosis. These findings indicated that iASPP may be a potential target for GC therapy. However, the specific mechanism whereby iASPP affects the biological behavior of tumor cells remains to be fully elucidated and its upstream and downstream factors remain to be identified. Further genetic studies are required to examine the signals of iASPP that are able to regulate the biological behavior of cancer cells.
